# Notes and News

**Published:** 1888-04-14

**Authors:** 


### Notes and News.

Collected and Communicated.

The subscriptions towards the new buildings of St. Mary's Hospital, Manchester, amount to £20,000.

During last year the Charing Cross Hospital has received in legacies and donations nearly £8,000.

Mrs. Ellerton has given a donation of £500 towards the nursing fund of the Leamington Provident Dispensary.

Mrs. Carr Burton has offered a sum of £2,500 for the purpose of completing the southern wing of the Brighton, Hove, and Preston Dispensary as a memorial of her late husband.

The late Miss Lowe has left a legacy of £3,283 to Bolton Infirmary, besides the reversion of a further sum of £4,500, to be received on the death of her nephew.

The widow and friends of the late Dr. Buck have built a handsome memorial chapel in the grounds of the Leicester Infirmary.

Nearly £150 was added to the Samaritan Fund of the Hospital for Children, Great Ormond Street, by the contributions, almost exclusively in pence, dropped into boxes in the out-patient department by the friends and relatives of the children under treatment.

On Saturday afternoon the Earl of Strafford laid the foundation-stone of a cottage hospital, which is about to be erected at Barnet, as a permanent memorial of her Majesty's Jubilee. There was read a message from the Queen, assuring the promoters of her sympathy with the movement, and conveying her royal command that the hospital, instead of being called "The Cottage Hospital for Barnet and District," as had been decided, should be termed The Victoria Cottage Hospital for Barnet and Neighbourhood.

The treasurer of the Berwick Infirmary is not a man who appreciates literature. He said, at the recent annual meeting, that he wished the people who send books to the infirmary would send old linen instead. They had a ward full of literature that was of no use, whereas all the old linen they had received was used. There is some justice in the complaint; but it does not arise from the fact that patients do not like books, but that many people make a practice of sending to the hospital just such old books and magazines as nobody at home cares to read.

A new wing has been built to the Mater Misericordiæ Hospital, Dublin.

The York Dispensary, which was founded in 1788, is celebrating its centenary this year. At a meeting held to celebrate this event, and to urge upon the public the duty of helping to support the dispensary, subscriptions to the amount of £837 were promised.

Dr. C. R. Drysdale, Senior Physician to the Metropolitan Free Hospital, thinks it likely that the epidemic of small-pox, which is now decimating Sheffield, may visit London and other towns, and strongly advises revaccination among adults as a preventive.

Scarlet fever has broken out on board the training ships *Shaftesbury* and *Exmouth*, lying off Grays. There have been 16 cases from the former and one from the latter removed to the Sanitary Asylum. The port sanitary authorities have the ships under strict surveillance, and it is hoped that no further outbreak will occur.

Dr. Whitcombe, the superintendent of the Winson Green Asylum, has recommended that a separate hospital for the insane should be erected by the Birmingham Town Council. He is of opinion that the treatment of the sick in asylums is far from satisfactory; indeed, he maintains that in some cases of mental disease removal to an asylum is the worst possible treatment.

The Derby Hospital for Sick Children appears to be a very well-managed institution. During the last year the number of in-patients increased by 27, and the corresponding increase of expenditure was only £37. A fire-extinguishing apparatus has been added to the hospital, in the use of which the nurses have been instructed. It is proposed to add an isolation ward to the hospital, which would cost £400. Towards this object Mr. Swingler has given £100. At the last annual meeting a silver inkstand was presented by the governors to Miss Cupiss, who for ten years has gratuitously filled the post of lady superintendent of the hospital.

That the Bradford Infirmary is economically managed may be inferred from one item in its accounts—that it received over £63 during last year from the sale of dripping. The work of the Infirmary goes on in a steadily progressing career of usefulness, and the lucid and interesting nature of the clinical report of its work deserves special mention. Like most other institutions, it is suffering from lack of funds, though it still keeps its expenditure within its income. The income of the Spencer Nursing Fund has now, however, become sufficient to justify the committee in separating the nursing from the domestic department, and a housekeeper and a superintendent of nurses have been elected to take charge of the two sections, a plan which will doubtless tend to greater efficiency in both.

At Luton Hospital they are discussing the advisability of having a Ladies' Committee. There seems to be but one opinion as to the usefulness of ladies being encouraged to interest themselves in the hospital; but it is feared that the existence of a female official body would cause jealousy and friction with the general committee. Such a fear is not wholly causeless; the existence of two bodies, with equal claims to authority, managing the same institution, would probably lead to some dissension. But as many departments of hospital management belong to matters in which women have more experience than men, the best plan would be to admit ladies to a place on the committee of management. They have rendered admirable service on school and parochial boards, and we are strongly of opinion that they would be equally useful on hospital committees.

